# Clinical, immunological, and radiological characteristics of pulmonary involvement in childhood-onset primary Sjögren’s syndrome: a single-center retrospective study

**DOI:** 10.3389/fimmu.2026.1943681

**Published:** 2026-10-06

**Authors:** Jiayun Ling, Tongyu Wu, Xiaohui Liu, Zhuqiang Wu, Caihui Yuan

**Affiliations:** 1 Department of Rheumatology and Immunology, Jiangxi Provincial Children’s Hospital, Nanchang Medical College, Nanchang, Jiangxi, China; 2 Department of Rheumatology and Immunology, Jiangxi Hospital Affiliated to Children’s Hospital of Chongqing Medical University, Nanchang, Jiangxi, China; 3 Norman Bethune First School of Clinical Medicine, Jilin University, Changchun, Jilin, China; 4 Radiology Department, Jiangxi Provincial Children’s Hospital, Nanchang Medical College, Nanchang, Jiangxi, China; 5 Radiology Department, Jiangxi Hospital Affiliated to Children’s Hospital of Chongqing Medical University, Nanchang, Jiangxi, China

**Keywords:** primary Sjögren's syndrome, childhood onset, interstitial lung disease, high-resolution computed tomography, lymphocyte subsets

## Abstract

**Objective:**

To describe pulmonary involvement in childhood-onset primary Sjögren’s syndrome (cSS).

**Methods:**

This retrospective study included 17 children with cSS diagnosed at Jiangxi Provincial Children’s Hospital between August 2021 and August 2025. Patients were classified by baseline chest high-resolution computed tomography (HRCT)as normal CT (n=8) or abnormal CT (n=9); among patients with abnormal CT, 5 had interstitial lung involvement. Clinical, laboratory, radiological, and outcome data were compared.

**Results:**

Among 17 children, 14 were female (82.4%), and the mean age at onset was 10.83 ± 3.12 years. Abnormal chest CT findings were detected in 9 patients (52.9%). Age at onset was younger in the abnormal CT group than in the normal CT group (9.37 ± 2.67 vs. 12.48 ± 2.88 years, *P* = 0.036), and was lowest in the interstitial lung involvement subgroup (8.30 ± 2.51 years, *P* = 0.022). This subgroup had higher total T and CD4+ T lymphocyte proportions and a lower B lymphocyte proportion *P* = 0.026, 0.027, and 0.023, respectively. Pleural thickening was the most common CT abnormality (77.8%), followed by ground-glass opacities and nodules >3 mm (33.3% each). Among the 5 patients with interstitial lung involvement, 3 met radiological criteria for ILD, comprising 2 with organizing pneumonia and 1 with nonspecific interstitial pneumonia. During follow-up, 3 of 7 patients with longitudinal imaging achieved complete radiological normalization, 2 improved, and 2 remained stable.

**Conclusion:**

Pulmonary abnormalities were common in children with cSS. Younger onset and aberrant T/B lymphocyte subsets showed exploratory associations with pulmonary involvement, which appeared mainly inflammatory and demonstrated favorable radiological evolution during follow-up.

## 1 Introduction

Primary Sjögren’s syndrome (pSS) is a chronic systemic autoimmune disease pathologically characterized by lymphocytic infiltration of the exocrine glands. It can involve multiple organs and cause irreversible functional impairment, substantially affecting long-term prognosis and quality of life (1, 2). Childhood-onset primary Sjögren’s syndrome (cSS) is rare (3), accounting for approximately 1% of all pSS cases in global multicenter cohorts (4, 5). Compared with adult-onset pSS, cSS shows greater clinical heterogeneity and often presents atypically (6). Classic sicca symptoms, such as dry mouth (xerostomia) and dry eyes (xerophthalmia), are uncommon as initial manifestations; instead, children more frequently present with recurrent parotid swelling and extraglandular multisystem involvement, which contributes to diagnostic difficulty and frequent delays in pediatric practice (3, 6, 7). Furthermore, the diagnosis of cSS remains challenging due to the absence of validated pediatric-specific classification criteria, with clinical and serological findings integrated with minor salivary gland biopsy forming the current diagnostic pathway (8).

Extraglandular involvement is a key determinant of disease severity, prognosis, and long-term quality of life in pSS (9). Among extraglandular manifestations, pulmonary involvement is one of the most common and serious complications (10). Adult cohort studies have shown that pSS-related pulmonary involvement predominantly manifests as interstitial lung disease (ILD), which is associated with increased all-cause mortality and is an important predictor of poor prognosis (11–13). Although the epidemiology, radiological patterns, prognostic factors, and treatment strategies for pulmonary involvement in adult pSS have been relatively well characterized (14, 15), available evidence in pediatric cSS remains limited. Current data are mainly derived from small case series and case reports (16), and the clinical phenotype, imaging characteristics, prognostic factors, and standardized early screening strategies for pulmonary involvement in children with cSS remain incompletely characterized.

This single-center retrospective descriptive study included children with cSS admitted to our hospital between August 2021 and August 2025. Demographic characteristics, clinical manifestations, laboratory findings, chest high-resolution computed tomography (HRCT) features, and short-term treatment outcomes were compared between patients with and without pulmonary involvement. This study aimed to characterize pulmonary involvement in children with cSS and explore potential associated factors, providing observational data that may support early recognition and clinical decision-making in this population.

## 2 Patients and methods

### 2.1 Study design and ethical approval

This single-center retrospective descriptive study was conducted at the Department of Rheumatology and Immunology, Jiangxi Provincial Children’s Hospital. Children with cSS who were first hospitalized and received a confirmed diagnosis between August 2021 and August 2025 were included. The study was approved by the Ethics Committee of Jiangxi Provincial Children’s Hospital (approval No. JXSETYY-YXKY-2026064) and conducted in accordance with the Declaration of Helsinki. Written informed consent from legal guardians was waived owing to the retrospective design, and all clinical data were anonymized.

### 2.2 Study population

Potentially eligible cases were identified from the electronic medical record system using ICD-10 code M35.0 (Sjögren’s syndrome). Of 20 children initially screened, 3 were excluded (2 with secondary Sjögren’s syndrome associated with systemic lupus erythematosus, and 1 with overlap syndrome), leaving 17 enrolled (**Figure 1**).

**Figure 1:**
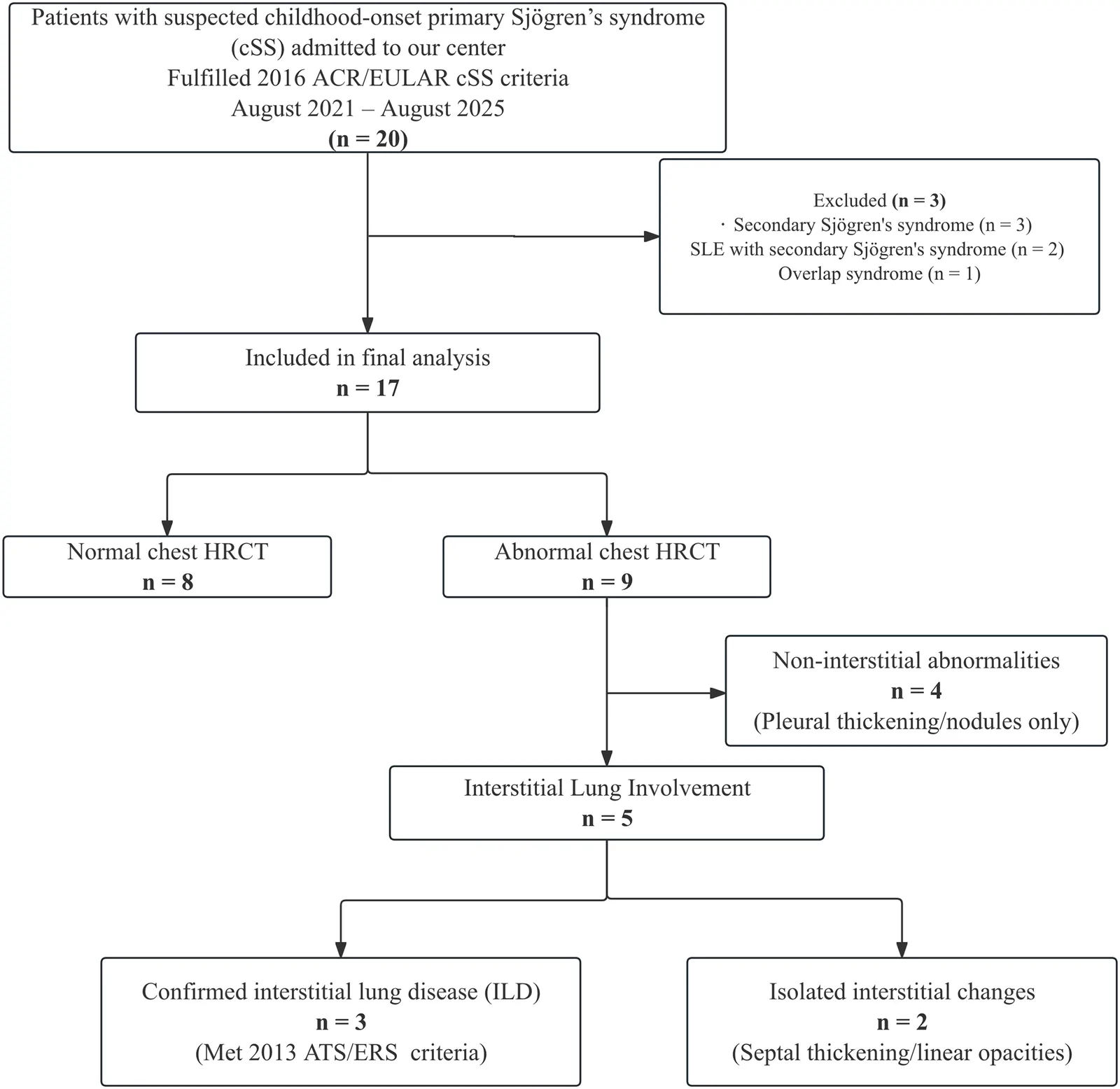
Flowchart of patient enrollment and study grouping.

Inclusion criteria were: (a) first hospitalization at our department with confirmed cSS diagnosis, baseline systemic evaluation, and completion of initial treatment; (b) age at disease onset ≤18 years and fulfillment of the 2016 American College of Rheumatology/European League Against Rheumatism (ACR/EULAR) classification criteria for primary Sjögren’s syndrome (17); (c) complete and traceable core clinical data, including demographic information, clinical manifestations, and baseline laboratory findings; and (d) baseline chest HRCT completed during hospitalization with imaging data sufficient for standardized radiological assessment. Clinical and radiological follow-up data for all included patients were censored on November 30, 2025.

The 2016 ACR/EULAR criteria remain the most widely applied diagnostic framework in pediatric practice, as no validated pediatric-specific classification criteria currently exist. Diagnostic components available in our cohort included anti-SSA/Ro testing, Schirmer’s test, ocular staining, and minor salivary gland biopsy; unstimulated salivary flow rate was not routinely performed.

Exclusion criteria were: (a) secondary Sjögren’s syndrome, including association with SLE, juvenile idiopathic arthritis, systemic sclerosis, or other systemic connective tissue diseases; (b) concomitant pulmonary diseases that could confound radiological assessment, including infectious pneumonia, pulmonary tuberculosis, bronchopulmonary dysplasia, acute exacerbation of bronchial asthma, or pulmonary infiltration by malignancy; (c) incomplete essential diagnostic data, baseline HRCT images, or follow-up data precluding analysis; and (d) loss to follow-up or follow-up duration less than 1 month.

### 2.3 Data collection

All data were extracted from the electronic medical record system of our hospital. Collected variables included demographic and clinical characteristics (sex, age at disease onset, age at diagnosis, disease duration before diagnosis, follow-up duration, initial symptoms, systemic manifestations, and baseline disease activity assessed using the EULAR Sjögren’s Syndrome Disease Activity Index (ESSDAI) (18)); laboratory parameters (complete blood count, inflammatory markers, liver and renal function, immunoglobulins, complement levels, autoantibody profiles, and peripheral blood lymphocyte subsets); and treatment regimens (systemic glucocorticoids, methylprednisolone pulse therapy, and immunosuppressive agents during initial hospitalization). Clinical variables are organized according to ESSDAI organ domains in **Table 1**.

**Table 1:** Clinical characteristics of 17 children with childhood-onset primary Sjögren’s syndrome.

Variables	Total (n=17)	Normal chest CT (n=8)	Abnormal chest CT group (n=9)	*P* value^a^	Interstitial lung involvement group (n=5)	*P* value^b^
Demographic characteristics						
Female sex, n (%)	14/17 (82.4)	8/8 (100.0)	6/9 (66.7)	0.206	4/5 (80.0)	0.385
Age at disease onset, years, mean ± SD	10.83 ± 3.12	12.48 ± 2.88	9.37 ± 2.67	0.036*	8.30 ± 2.51	0.022△
Disease duration before diagnosis, weeks, median [IQR]	6.43 [2.86, 17.14]	5.43 [2.86, 22.50]	8.57 [5.00, 10.00]	0.772	5.00 [2.00, 6.43]	0.509
Follow-up duration, months, median [IQR]	11.00 [3.00, 21.00]	16.00 [5.75, 27.50]	7.00 [3.00, 18.00]	0.469	12.00 [3.00, 18.00]	0.346
Initial symptoms, n (%)						
Fever	2/17 (11.8)	2/8 (25.0)	0/9 (0.0)	0.206	0/5 (0.0)	0.487
Fatigue	1/17 (5.9)	1/8 (12.5)	0/9 (0.0)	0.471	0/5 (0.0)	1.000
Limb tremor	1/17 (5.9)	1/8 (12.5)	0/9 (0.0)	0.471	0/5 (0.0)	1.000
Arthralgia	3/17 (17.6)	1/8 (12.5)	2/9 (22.2)	1.000	2/5 (40.0)	0.510
Skin rash	5/17 (29.4)	1/8 (12.5)	4/9 (44.4)	0.294	2/5 (40.0)	0.510
Thrombocytopenia	4/17 (23.5)	1/8 (12.5)	3/9 (33.3)	0.576	1/5 (20.0)	1.000
Anemia	1/17 (5.9)	1/8 (12.5)	0/9 (0.0)	0.471	0/5 (0.0)	1.000
Clinical manifestations, n (%)						
Fever	4/17 (23.5)	2/8 (25.0)	2/9 (22.2)	1.000	2/5 (40.0)	1.000
Fatigue	7/17 (41.2)	5/8 (62.5)	2/9 (22.2)	0.153	1/5 (20.0)	0.266
Superficial lymphadenopathy	13/17 (76.5)	6/6 (100.0)	7/9 (77.8)	0.486	5/5 (100.0)	1.000
Xerostomia	4/17 (23.5)	3/8 (37.5)	1/9 (11.1)	0.294	1/5 (20.0)	1.000
Xerophthalmia	7/17 (41.2)	4/8 (50.0)	3/9 (33.3)	0.637	2/5 (40.0)	1.000
Parotid enlargement	4/17 (23.5)	2/8 (25.0)	2/9 (22.2)	1.000	1/5 (20.0)	1.000
Abnormal parotid ultrasound	9/17 (52.9)	4/8 (50.0)	5/9 (55.6)	1.000	4/5 (80.0)	0.565
Motor system involvement	5/17 (29.4)	2/8 (25.0)	3/9 (33.3)	1.000	3/5 (60.0)	0.293
Purpuric rash	3/17 (17.6)	1/8 (12.5)	2/9 (22.2)	1.000	1/5 (20.0)	1.000
Erythematous rash	6/17 (35.3)	2/8 (25.0)	4/9 (44.4)	0.620	2/5 (40.0)	1.000
Cough	2/17 (11.8)	0/8 (0.0)	2/9 (22.2)	0.471	1/5 (20.0)	0.385
Dyspnea	0/17 (0.0)	0/8 (0.0)	0/9 (0.0)	1.000	0/5 (0.0)	1.000
Renal tubular acidosis	1/17 (5.9)	1/8 (12.5)	0/9 (0.0)	0.471	0/5 (0.0)	1.000
Myalgia/muscle weakness	1/17 (5.9)	1/8 (12.5)	0/9 (0.0)	0.471	0/5 (0.0)	1.000
Elevated creatine kinase	1/17 (5.9)	1/8 (12.5)	0/9 (0.0)	0.471	0/5 (0.0)	1.000
Central nervous system involvement	2/17 (11.8)	2/8 (25.0)	0/9 (0.0)	0.206	0/5 (0.0)	0.487
Anemia	9/17 (52.9)	4/8 (50.0)	5/9 (55.6)	1.000	3/5 (60.0)	1.000
Thrombocytopenia	5/17 (29.4)	2/8 (25.0)	3/9 (33.3)	1.000	1/5 (20.0)	1.000
Hypocomplementemia	7/17 (41.2)	2/8 (25.0)	5/9 (55.6)	0.335	4/5 (80.0)	0.103
Hypergammaglobulinemia	10/17 (58.8)	4/8 (50.0)	6/9 (66.7)	0.637	4/5 (80.0)	0.565
Diagnostic criteria components						
Minor salivary gland biopsy ≥Chisholm-Mason grade III, n (%)	16/17 (94.1)	8/8 (100.0)	8/9 (88.9)	1.000	4/5 (80.0)	0.385
Abnormal ocular examination, n (%)	7/17 (41.2)	3/8 (37.5)	4/9 (44.4)	1.000	2/5 (40.0)	1.000
Anti-SSA/Ro positivity, n (%)	16/17 (94.1)	8/8 (100.0)	8/9 (88.9)	1.000	4/4 (100.0)	0.385
Anti-SSB/La positivity, n (%)	12/17 (70.6)	7/8 (87.5)	5/9 (55.6)	0.294	2/5 (40)	0.217
Anti-Ro52 positivity, n (%)	11/17 (64.7)	6/8 (75.0)	5/9 (55.6)	0.620	3/5 (60)	1.000

^a^Comparison between the normal chest CT group and the abnormal chest CT group. ^b^Comparison between the normal chest CT group and the interstitial lung involvement group. *Compared with the normal chest CT group, *P* < 0.05. △Compared with the normal chest CT group, *P* < 0.05;Minor salivary gland biopsy was performed in all 17 patients. Biopsy abnormalities are defined as ≥Chisholm-Mason grade III, corresponding to focal lymphocytic sialadenitis with a focus score ≥1 focus/4mm² (i.e., at least one aggregate of ≥50 lymphocytes per 4mm² of glandular tissue), as specified in the 2016 ACR/EULAR classification criteria for Sjögren’s syndrome.

### 2.4 Radiological assessment

Baseline chest HRCT was systematically performed as part of the standardized systemic evaluation for all newly diagnosed children with cSS at our center, regardless of respiratory symptoms. All scans were acquired using a Philips Ingenuity 64-slice CT system with high-spatial-frequency reconstruction and a slice thickness of 0.5 mm. Two radiologists with more than 5 years of experience in pediatric chest imaging independently reviewed all images, blinded to the patients’ clinical data.

Radiological assessment comprised five dimensions: (a) identification of all abnormal chest CT findings; (b) characterization of lesion distribution patterns; (c) determination of pulmonary interstitial involvement; (d) severity grading using the Warrick score (range 0–30, with higher scores indicating more severe interstitial disease); and (e) classification of ILD subtypes based on the 2013 ATS/ERS international multidisciplinary classification of idiopathic interstitial pneumonias (19). The Warrick score was used as a pragmatic semi-quantitative measure of interstitial abnormality severity, given the absence of a validated pediatric-specific scoring system.

The following hierarchical operational definitions were applied: (a) Abnormal chest CT was defined as any identifiable parenchymal, pleural, or airway abnormality on baseline HRCT, excluding technical artifacts and normal variants. (b) Interstitial lung involvement was defined as interstitial abnormalities (ground-glass opacities, linear opacities, interlobular septal thickening, subpleural lines, reticular opacities, traction bronchiectasis, or consolidation with interstitial predominance) involving at least 5% of any lung zone, adapted from the Fleischner Society definition of interstitial lung abnormalities. (c) Definite ILD was diagnosed when interstitial lung involvement was accompanied by at least one of the following: HRCT findings consistent with a specific ILD pattern according to the 2013 ATS/ERS classification (NSIP, OP, UIP, or LIP); respiratory symptoms or signs attributable to ILD; or abnormal pulmonary function tests.

For patients with fever and cough, empirical antibiotic therapy was administered; those whose respiratory symptoms persisted despite treatment and in whom no pathogenic organism was identified were considered to have autoimmune-related pulmonary involvement rather than infection.

Interobserver agreement was assessed using Cohen’s kappa for categorical variables and the intraclass correlation coefficient (ICC) for the Warrick score. Discrepant interpretations were adjudicated by a senior pediatric chest radiologist with more than 10 years of diagnostic experience. Follow-up HRCT examinations were performed using the identical scanning protocol and reviewed following the same standardized procedure. Post-treatment radiological changes were categorized as complete normalization, continued improvement, stable disease, or disease progression.

### 2.5 Statistical analysis

Statistical analyses were performed using IBM SPSS Statistics version 29.0. Continuous variables were assessed for normality using the Shapiro–Wilk test. Normally distributed variables are presented as mean ± standard deviation and compared between groups using the independent-samples t-test; non-normally distributed variables are presented as median and interquartile range (IQR) and compared using the Mann–Whitney U test. Categorical variables are expressed as frequencies and percentages, and between-group comparisons were performed using Pearson’s chi-square test or Fisher’s exact test when the expected frequency was less than 5. All statistical tests were two-sided, with a significance level of α = 0.05. Given the small sample size and the descriptive, exploratory nature of this study, no adjustment for multiple comparisons was applied, and multivariable regression analysis was not performed. All between-group comparisons, including parametric tests in small subgroups, should be interpreted as hypothesis-generating.

## 3 Results

### 3.1 Baseline enrollment characteristics

A total of 17 treatment-naïve children with cSS who fulfilled the 2016 ACR/EULAR classification criteria were included in this study. Diagnostic characterization demonstrated minor salivary gland biopsy ≥Chisholm-Mason grade III in 94.1% (16/17). **Figure 1** summarizes patient enrollment and study grouping. Based on baseline chest HRCT findings, the patients were divided into a normal chest CT group (n = 8) and an abnormal chest CT group (n = 9). Among the patients with abnormal chest CT findings, 5 showed evidence of interstitial lung involvement, of whom 3 met criteria for definite ILD, including 2 with radiological features consistent with organizing pneumonia and 1 with features consistent with nonspecific interstitial pneumonia.

### 3.2 Comparison of demographic and clinical characteristics

Baseline demographic and clinical characteristics are summarized in **Table 1**. Among the 17 patients, 14 were female (82.4%), and the mean age at disease onset was 10.83 ± 3.12 years. Age at onset was significantly younger in the abnormal chest CT group than in the normal chest CT group (9.37 ± 2.67 vs. 12.48 ± 2.88 years, *P* = 0.036). The interstitial lung involvement subgroup also showed a significantly younger age at onset than the normal chest CT group (8.30 ± 2.51 years, *P* = 0.022). During the disease course, cough occurred in 11.8% (2/17, both in the abnormal CT group), and no patient reported dyspnea. Notable features included superficial lymphadenopathy (86.7%), hypergammaglobulinemia (58.8%), and hypocomplementemia (41.2%). No other between-group differences reached statistical significance in disease duration before diagnosis, follow-up duration, or clinical variables organized by ESSDAI domains (all *P* ***>*** 0.05).

### 3.3 Comparison of laboratory parameters

Laboratory characteristics are summarized in **Table 2**. Compared with the normal chest CT group, the interstitial lung involvement subgroup had significantly higher percentages of total T lymphocytes and CD4+ T lymphocytes and a significantly lower percentage of B lymphocytes (*P* = 0.026, 0.027, and 0.023, respectively). No significant differences were observed between groups in complete blood count, inflammatory markers, liver and renal function, immunoglobulin levels, complement levels, or autoantibody profiles (all *P* > 0.05).

**Table 2:** Laboratory characteristics of 17 children with childhood-onset primary Sjögren’s syndrome.

Parameters	Normal chest CT group (n = 8)	Abnormal chest CT group (n = 9)	*P* value^a^	Interstitial lung involvement group (n = 5)	P value^b^
WBC, ×10^9^/L, median [IQR]	5.94 [5.30, 6.51]	6.25 [5.62, 8.54]	0.441	8.54 [7.74, 9.07]	0.057
Neutrophils, ×10^9^/L, median [IQR]	3.46 [3.10, 4.52]	3.06 [2.60, 4.16]	0.700	4.16 [3.72, 6.80]	0.380
Lymphocytes, ×10^9^/L, median [IQR]	1.65 [1.42, 1.82]	1.86 [1.37, 2.53]	0.462	2.02 [1.07, 3.23]	1.000
Hemoglobin, g/L, mean ± SD	113.00 ± 19.15	113.11 ± 12.10	0.989	113.40 ± 14.45	0.969
Platelets, ×10^9^/L, mean ± SD	209.00 ± 118.64	212.67 ± 160.50	0.958	276.00 ± 156.76	0.398
CRP, mg/L, median [IQR]	2.98 [0.42, 9.32]	0.20 [0.10, 8.31]	0.698	0.80 [0.20, 8.31]	0.883
Fibrinogen, g/L, median [IQR]	2.41 [2.28, 2.77]	2.29 [2.02, 2.93]	0.562	2.18 [2.04, 2.94]	0.395
ALT, U/L, median [IQR]	26.50 [18.75, 29.50]	18.18 [11.00, 67.00]	1.000	11.00 [10.00, 18.18]	0.188
AST, U/L, median [IQR]	31.50 [27.68, 34.50]	39.00 [29.11, 58.00]	0.441	32.00 [29.11, 39.00]	0.660
CK, U/L, median [IQR]	73.50 [36.00, 134.12]	73.78 [41.30, 98.00]	0.847	73.78 [41.30, 98.00]	0.884
LDH, U/L, median [IQR]	218.50 [194.50, 303.25]	260.49 [245.28, 290.00]	0.336	260.49 [253.00, 290.00]	0.242
Serum potassium, mmol/L, median [IQR]	3.71 [3.58, 4.02]	3.87 [3.74, 4.04]	0.385	3.94 [3.83, 4.02]	0.443
IgG, g/L, mean ± SD	16.53 ± 4.97	16.93 ± 4.94	0.869	17.64 ± 5.43	0.711
C3, g/L, mean ± SD	1.10 ± 0.32	1.10 ± 0.32	0.975	0.99 ± 0.32	0.577
C4, g/L, mean ± SD	0.19 ± 0.07	0.18 ± 0.07	0.631	0.15 ± 0.07	0.262
RF, IU/mL, mean ± SD	68.18 ± 54.29	80.49 ± 88.07	0.746	116.16 ± 104.39	0.308
Total T lymphocytes, %, mean ± SD	65.88 ± 7.58	70.61 ± 10.33	0.305	76.06 ± 5.59	0.026*
CD4^+^ T lymphocytes, %, mean ± SD	31.52 ± 8.19	39.30 ± 9.78	0.098	44.61 ± 10.33	0.027*
CD8^+^ T lymphocytes, %, mean ± SD	28.68 ± 6.76	25.78 ± 9.64	0.490	27.31 ± 10.83	0.783
B lymphocytes, %, mean ± SD	27.58 ± 8.80	20.89 ± 9.24	0.148	15.97 ± 5.30	0.023*
NK cells, %, mean ± SD	5.87 ± 2.55	7.59 ± 4.14	0.325	6.86 ± 3.49	0.562
ESSDAI score, mean ± SD	20.62 ± 8.02	15.00 ± 5.02	0.099	17.40 ± 5.32	0.446

^a^Comparison between the normal chest CT group and the abnormal chest CT group. ^b^Comparison between the normal chest CT group and the interstitial lung involvement group. *P < 0.05 compared with the normal chest CT group.

WBC, white blood cell count; CRP, C-reactive protein; ALT, alanine aminotransferase; AST, aspartate aminotransferase; CK, creatine kinase; LDH, lactate dehydrogenase; Ig, immunoglobulin; C3, complement component 3; C4, complement component 4; RF, rheumatoid factor; NK, natural killer; ESSDAI, EULAR Sjögren’s Syndrome Disease Activity Index; IQR, interquartile range; SD, standard deviation.

### 3.4 Radiological characteristics of patients with abnormal chest CT findings

All HRCT studies were independently reviewed by two pediatric radiologists. Interobserver agreement was excellent for the semiquantitative Warrick score (ICC = 0.95) and substantial for ILD pattern classification (Cohen’s κ = 0.62), with disagreements adjudicated by a senior radiologist.

Chest HRCT findings in patients with abnormal baseline CT results are summarized in **Table 3**. Among the 9 patients with abnormal baseline chest CT findings, pleural thickening was the most frequently observed HRCT abnormality, present in 77.8% of patients (7/9). Ground-glass opacities and pulmonary nodules >3 mm were each identified in 33.3% of patients (3/9). Linear opacities, interlobular septal thickening/subpleural lines, and pulmonary consolidation were each observed in 22.2% of patients (2/9). Less frequently observed findings included reticular opacities, consolidation with the reversed halo sign, consolidation with air bronchograms, and traction bronchiectasis, each present in 11.1% of patients (1/9).

**Table 3:** Chest HRCT findings in children with childhood-onset primary Sjögren’s syndrome.

Category	Specific findings	Results
Chest HRCT findings (*n* = 9)	Pleural thickening, n (%)	7 (77.8)
	Ground-glass opacities, n (%)	3 (33.3)
	Pulmonary nodules > 3 mm, n (%)	3 (33.3)
	Linear opacities, n (%)	2 (22.2)
	Interlobular septal thickening/subpleural lines, n (%)	2 (22.2)
	Pulmonary consolidation, n (%)	2 (22.2)
	Reticular opacities, n (%)	1 (11.1)
	Consolidation with reversed halo sign, n (%)	1 (11.1)
	Consolidation with air bronchograms, n (%)	1 (11.1)
	Traction bronchiectasis, n (%)	1 (11.1)
	Warrick score, median [IQR]	5 [1.5, 8.0]
Interstitial lung disease (n = 3)	Nonspecific interstitial pneumonia (NSIP), n (%)	1 (33.3)
	Organizing pneumonia, n (%)	2 (66.7)

Among the 5 patients with interstitial lung involvement, 2 showed limited septal thickening and linear opacities that did not meet criteria for definite ILD. The remaining 3 patients met criteria for definite ILD, including 2 with radiological features consistent with organizing pneumonia and 1 with features consistent with nonspecific interstitial pneumonia. Baseline ventilatory function tests were within normal limits in 2 of the 3 patients with definite ILD. The median Warrick score among patients with interstitial lung involvement was 5 (IQR, 1.5–8.0).

### 3.5 Treatment regimens and radiological outcomes

Initial treatment regimens are summarized in **Table 4**. Among the 17 patients, systemic glucocorticoids were administered to 16 patients (94.1%), hydroxychloroquine to 14 (82.4%), and mycophenolate mofetil to 12 (70.6%). Systemic glucocorticoids were used in all patients in both the abnormal chest CT group and the interstitial lung involvement subgroup, and hydroxychloroquine was administered to all patients in the interstitial lung involvement subgroup. Treatment distributions did not differ significantly between groups (all *P* > 0.05).

**Table 4:** Distribution of initial treatment regimens in children with childhood-onset primary Sjögren’s syndrome.

Treatment	Total (n = 17)	Normal chest CT group (n = 8)	Abnormal chest CT group (n = 9)	*P* value^a^	Interstitial lung involvement group (n = 5)	*P* value^b^
Systemic glucocorticoids, n (%)	16 (94.1)	7 (87.5)	9 (100.0)	0.471	5 (100.0)	>0.999
Methylprednisolone pulse therapy, n (%)	1 (5.9)	1 (12.5)	0 (0.0)	0.471	0 (0.0)	>0.999
Mycophenolate mofetil (MMF), n (%)	12 (70.6)	5 (62.5)	7 (77.8)	0.620	4 (80.0)	>0.999
Methotrexate (MTX), n (%)	5 (29.4)	3 (37.5)	2 (22.2)	0.620	1 (20.0)	>0.999
Hydroxychloroquine, n (%)	14 (82.4)	7 (87.5)	7 (77.8)	>0.999	5 (100.0)	>0.999

^a^Comparison between the normal chest CT group and the abnormal chest CT group. ^b^Comparison between the normal chest CT group and the interstitial lung involvement group.

Among the 9 patients with abnormal baseline chest CT findings, 2 underwent only baseline CT evaluation and had no follow-up imaging data available. The remaining 7 patients completed at least two longitudinal CT assessments, and their radiological trajectories are presented in a swimmer plot (**Figure 2**). Moreover, radiological evolution was generally favorable during follow-up. Among the 7 patients with available longitudinal imaging, 3 achieved complete radiological normalization, 2 showed radiological improvement, and 2 remained radiologically stable. One patient experienced transient radiological worsening; however, the findings remained stable during subsequent follow-up after adjustment of the immunosuppressive regimen. Representative serial chest HRCT changes are presented in **Figures 3**–**5**.

**Figure 2:**
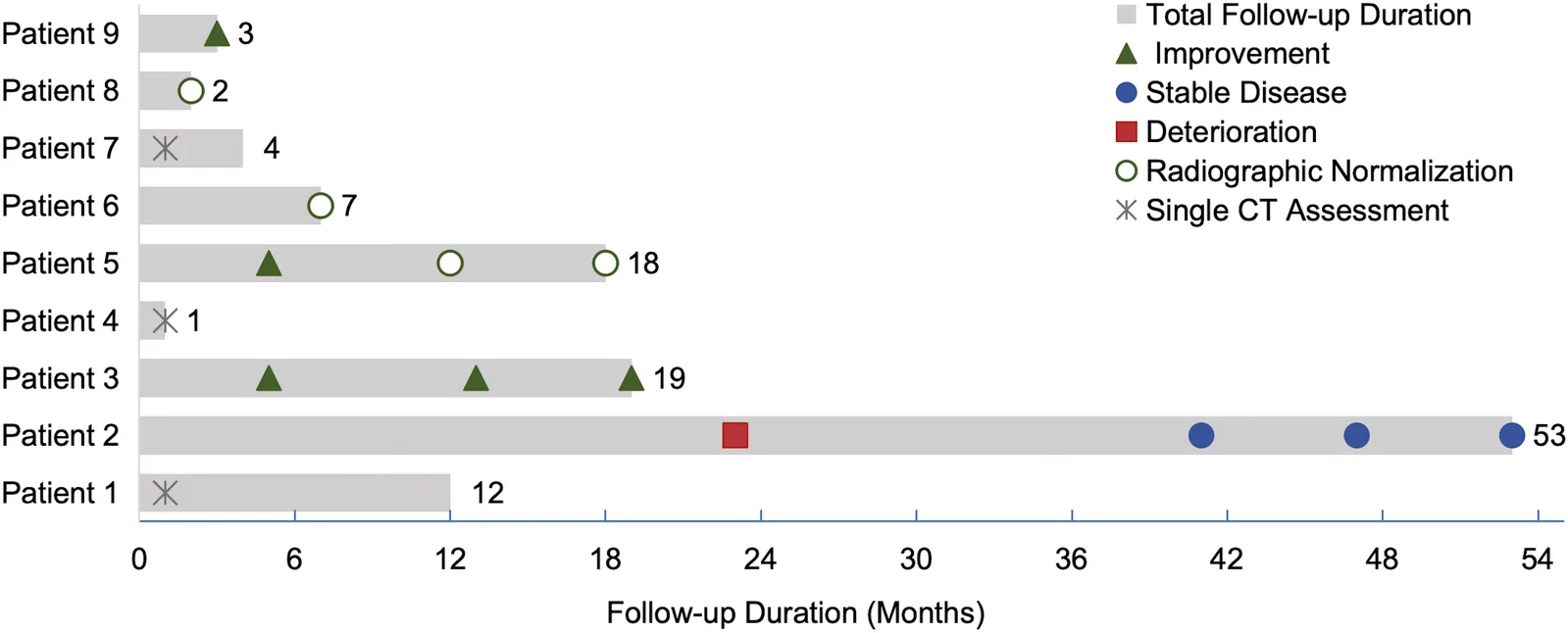
Swimmer plot illustrating longitudinal chest high-resolution computed tomography (HRCT) outcomes in patients with childhood-onset primary Sjögren’s syndrome (cSS) and pulmonary involvement. Each bar represents one patient, with length indicating follow-up duration (months).

**Figure 3:**
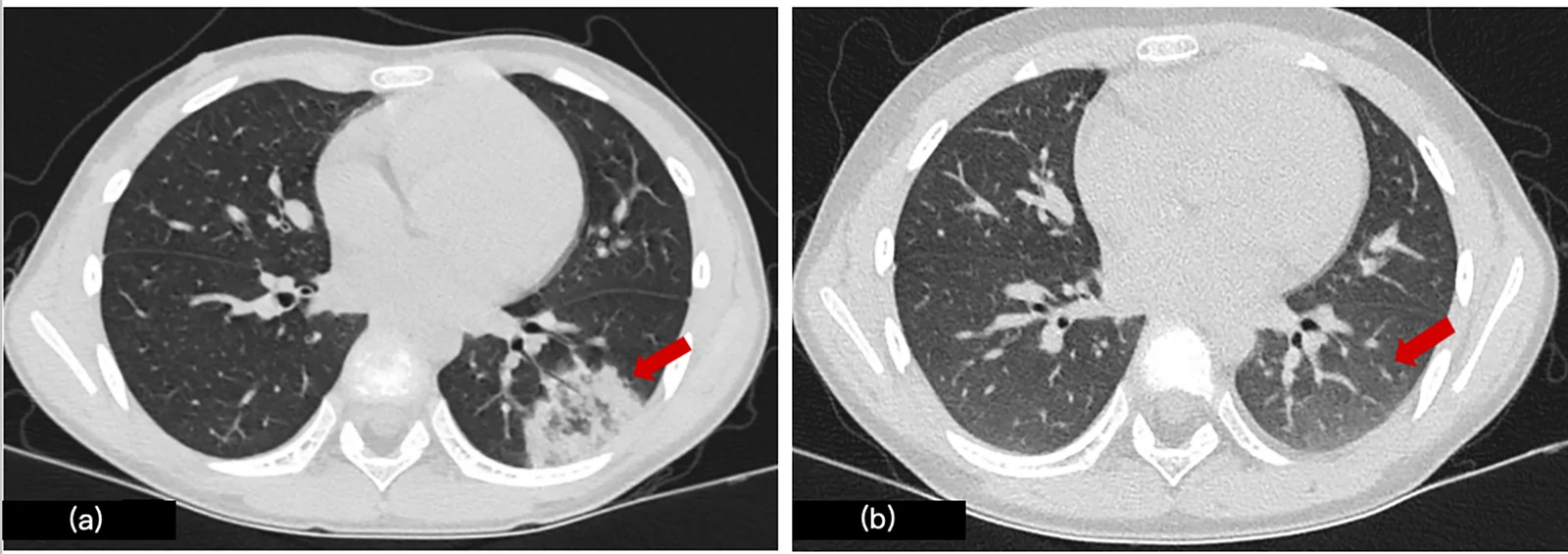
Serial HRCT images of cSS-associated organizing pneumonia in a 12-year-1-month-old male. **(a)** Baseline axial HRCT showing diffuse subpleural ground-glass opacities, traction bronchiectasis, pleural thickening, linear opacities, and the reversed halo sign (Warrick score = 9). **(b)** Two-month follow-up after prednisone (2 mg/kg/day, tapered), mycophenolate mofetil, and hydroxychloroquine showed near-complete lesion resolution (Warrick score = 0).

**Figure 4:**
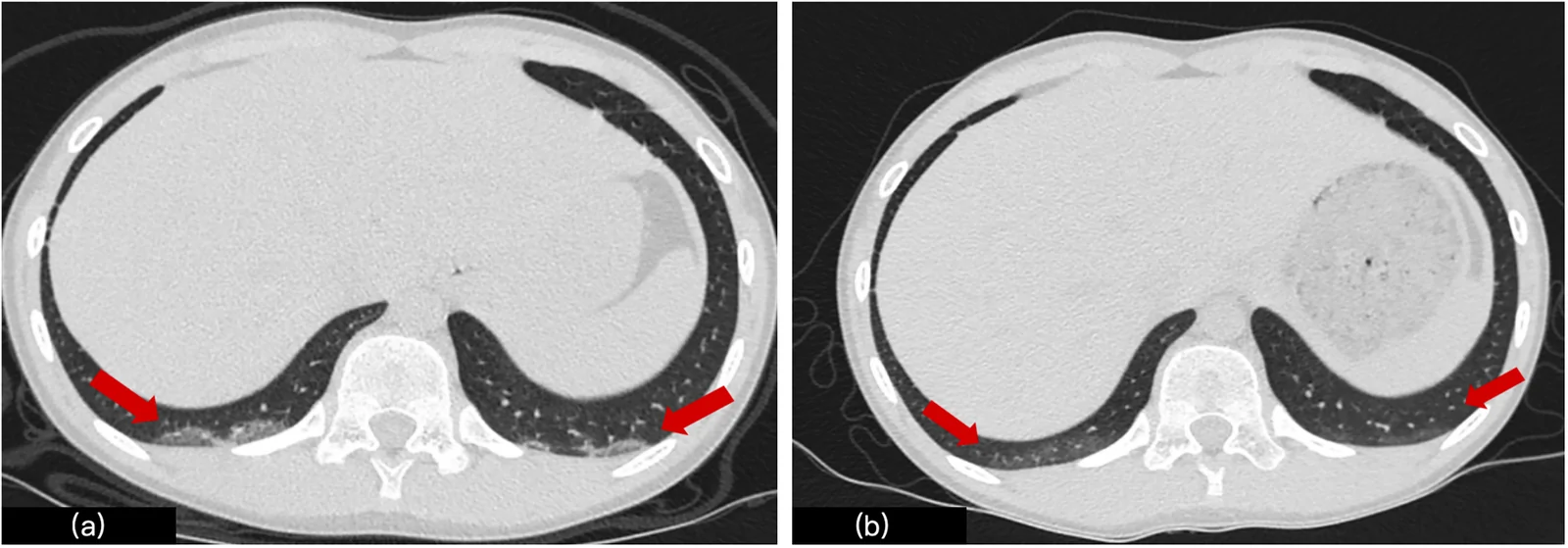
Serial HRCT images of cSS-associated organizing pneumonia in a 6-year-7-month-old female. **(a)** Baseline axial HRCT showing patchy subpleural ground-glass opacities, consolidation with air bronchograms, the reversed halo sign, and ipsilateral pleural thickening (Warrick score = 5). **(b)** Five-month follow-up after low-dose prednisone (0.25 mg/kg/day, tapered), mycophenolate mofetil, and hydroxychloroquine showing substantial radiological improvement (Warrick score = 2).

**Figure 5:**
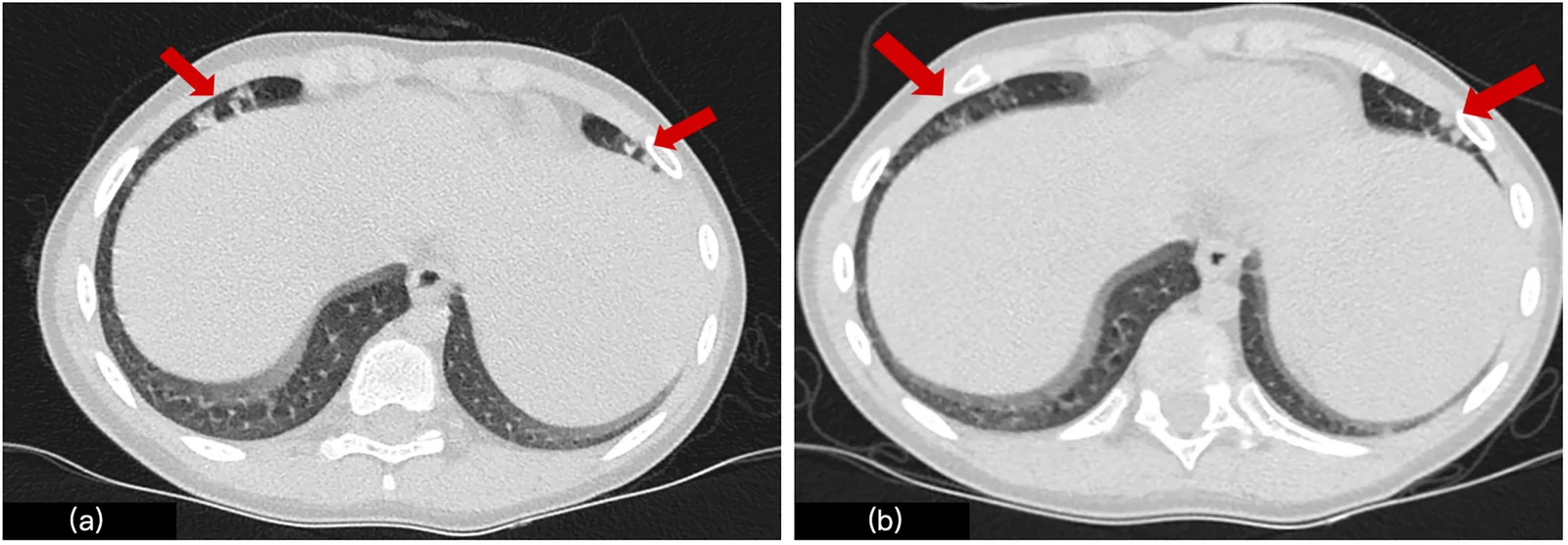
Serial HRCT images of cSS-associated nonspecific interstitial pneumonia in a 6-year-11-month-old female. **(a)** Baseline axial HRCT showing patchy subpleural ground-glass opacities, consolidation, fine reticular opacities, and bilateral pleural thickening (Warrick score = 9). **(b)** Three-month follow-up after prednisone (1.4 mg/kg/day, tapered), methotrexate, and hydroxychloroquine showing partial radiological improvement (Warrick score = 5).

## 4 Discussion

The clinical characteristics of pulmonary involvement in children with cSS remain incompletely characterized (16). Current evidence is largely limited to case reports and small case series, and cohort-based studies focusing on pulmonary involvement in this population are scarce. In this single-center retrospective study, 52.9% of children with cSS demonstrated abnormal chest CT findings. Pleural thickening was the most frequently observed radiological finding, followed by ground-glass opacities and pulmonary nodules. Patients with abnormal chest CT findings were younger at disease onset, and those with interstitial lung involvement showed aberrant T- and B-lymphocyte subset proportions. Moreover, radiological outcomes was generally favorable during follow-up. These preliminary observations, though hypothesis-generating given the small sample size, may contribute to improved clinical recognition of pulmonary involvement in children with cSS.

Pulmonary involvement in adult-onset primary Sjögren’s syndrome (pSS) has been extensively characterized. A 2020 systematic review reported that interstitial lung disease (ILD) represents the dominant phenotype of pulmonary involvement in adult pSS, with an overall prevalence of approximately 13% (12). Systematic chest high-resolution computed tomography (HRCT) screening of asymptomatic pSS patients has identified pulmonary abnormalities in 34% to 50% of cases (20). A 2025 large-scale retrospective study including 558 patients with pSS-ILD demonstrated that the most common HRCT pattern in adult pSS-ILD was nonspecific interstitial pneumonia (NSIP, 67.9%), followed by usual interstitial pneumonia (UIP, 18.8%), with organizing pneumonia (OP) being the least frequent (0.9%) (15). Adult studies have also identified age >50 years as an independent predictor of ILD in patients with pSS, suggesting that ILD risk increases with age, possibly owing to age-related pulmonary immune dysregulation and degenerative changes in lung tissue (13, 21, 22). In contrast to findings reported in adult populations, this study observed that children with cSS and abnormal chest CT findings were younger at disease onset. The mean age at onset in the interstitial lung involvement subgroup was only 8.30 years, suggesting that younger age at onset may be a hypothesis-generating signal associated with chest CT abnormalities in children with cSS. This finding is consistent with the study by Kılbaş et al. (6), which showed that children with prepubertal-onset cSS (defined as disease onset at ≤10 years of age) had higher systemic disease activity and a significantly increased risk of extraglandular involvement compared with those with pubertal-onset disease. Diagnostic delay may be associated with more extensive extraglandular involvement in younger children with cSS. As reported in previous studies, children with cSS, particularly those with early onset, frequently lack classic sicca symptoms at initial presentation, which has been associated with significant diagnostic delays (23–25). This delayed recognition may allow persistent autoimmune inflammation to progress, potentially resulting in more severe and extensive systemic manifestations, including life-threatening pulmonary complications such as diffuse alveolar hemorrhage (23).

During childhood, lung tissue is still developing, and the alveolar epithelium and interstitium may be more susceptible to autoimmune-mediated inflammation (26). In the setting of systemic immune activation, early pulmonary involvement may occur more readily. Accordingly, baseline chest HRCT screening may be considered in younger children with cSS, particularly those with high disease activity, although prospective validation is needed.

The present study found that the radiological manifestations of pulmonary involvement in children with cSS differ significantly from those observed in adult pSS. Adult pSS-ILD is characterized predominantly by fibrosis-related radiological signs (27). Ground-glass opacities and reticular opacities are the most common findings, indicating a predominance of inflammation and early fibrosis, while honeycombing indicates advanced fibrosis and is associated with a poor prognosis (15). In contrast, the most frequent CT abnormality in our cohort was pleural thickening, followed by ground-glass opacities and pulmonary nodules. Pleural thickening is recognized as a clinically relevant manifestation of pSS-related pulmonary involvement. A recent study by Ferro et al. demonstrated that pleural irregularities on lung ultrasound correlate with HRCT-defined ILD, with a postero-inferior score cutoff of 15 achieving 86.6% sensitivity and 84.2% specificity for ILD diagnosis (28). Pulmonary nodules were present in one-third of patients with abnormal CT findings and warrant attention in pSS. In a retrospective series of 41 adult pSS patients with biopsy-proven pulmonary nodules, Casal Moura et al. reported that more than half were malignant, predominantly non-Hodgkin lymphoma (39%) and lung carcinoma (27%) (29). In childhood-onset cSS, where both malignancy and advanced fibrotic lung disease are uncommon, the clinical significance of these radiological findings remains to be established.

Fibrosis-related abnormalities were observed in only one patient, and no cases of honeycombing or other end-stage fibrotic changes were identified. Consistent with previously reported pediatric cases of pSS-associated ILD, these findings suggest that early pulmonary involvement in cSS is predominantly inflammatory rather than fibrotic (16, 30, 31). The low frequency of fibrotic abnormalities may be related to the developmental characteristics of pediatric lung tissue, as well as earlier clinical recognition and intervention.

Currently, there are no specific treatment data for cSS-ILD, and treatment regimens are extrapolated from experience with adult pSS-ILD, with glucocorticoids combined with immunosuppressive agents forming the core therapeutic approach (16). Cyclophosphamide (CYC) and mycophenolate mofetil (MMF) are the most commonly used immunosuppressive agents, and their combination can reduce cumulative glucocorticoid doses and improve therapeutic outcomes (32). Studies have shown that organizing pneumonia (OP) responds well to immunosuppressive therapy, with rapid improvement in pulmonary function (33). In the present study, all children with abnormal chest CT findings received immunosuppressive therapy, albeit with heterogeneous regimens. Among the 7 patients with longitudinal imaging follow-up, 2 achieved complete radiological normalization, 3 showed continued improvement, and 2 remained stable. These observed radiological trajectories, though limited by small sample size and heterogeneous treatment regimens, are consistent with the predominantly inflammatory nature of early cSS-related pulmonary lesions. Early recognition of pulmonary involvement may facilitate timely clinical management, although prospective controlled studies are needed to determine optimal treatment strategies.

Although limited by the small interstitial lung involvement subgroup (n = 5), the observed lymphocyte subset alterations align with current understanding of autoimmune-mediated interstitial inflammation. In this study, children in the interstitial lung involvement subgroup showed aberrant lymphocyte subset proportions. Compared with the normal chest CT group, they had higher percentages of total T lymphocytes and CD4+ T lymphocytes, and a lower percentage of B lymphocytes. These findings suggest that abnormal activation of CD4+ T cells may play a central role in cSS-related interstitial lung involvement. Previous studies have demonstrated that abnormal effector functions of CD4+ T cells are crucial in the pathogenesis of Sjögren’s syndrome and systemic autoimmune responses (34). Single-cell RNA sequencing studies have further identified CD4+ effector memory T cells enriched for interferon-related genes as potential key driver cells in pediatric Sjögren’s syndrome (35). Previous studies have also shown that abnormal expansion of CD4+ T cells and Th1/Th17 proinflammatory polarization may directly contribute to alveolar epithelial injury, fibroblast activation, and interstitial inflammation in ILD (36, 37). The degree of CD4+ T-cell infiltration in lung tissue has been reported to correlate positively with ILD severity (37). A study evaluating the diagnostic value of lung ultrasound in connective tissue disease-associated ILD showed that lung ultrasound scores in SS-associated ILD were significantly correlated with HRCT findings, and higher scores were closely associated with increased peripheral blood CD4+ T-cell levels (38). These findings further support the role of CD4+ T-cell-mediated immune inflammation in the pathogenesis of pSS-associated ILD. Therefore, the observed lymphocyte subset alterations may represent exploratory immunological associations that, if validated in larger cohorts, could inform risk stratification for interstitial lung involvement and provide a basis for hypothesis-generating targeted immunotherapeutic strategies.

This study has several limitations. First, the single-center retrospective design and small sample size limited statistical power; multivariable regression could not be performed, and the unadjusted lymphocyte subset associations should be viewed as exploratory. Second, variable follow-up duration and incomplete longitudinal pulmonary function data precluded assessment of long-term functional outcomes, and the Warrick score, applied here as a semiquantitative tool, has limited validation in pediatric ILD cohorts. Finally, the study was restricted to clinical phenotyping without mechanistic experiments. Future multicenter prospective studies are needed to confirm these observations and elucidate the immune mechanisms underlying pulmonary involvement in cSS.

## 5 Conclusion

In this single-center cohort, 52.9% of children with cSS demonstrated abnormal chest CT findings. Younger age at disease onset was associated with chest CT abnormalities and may represent a hypothesis-generating signal for pulmonary involvement in this population. Children with interstitial lung involvement showed aberrant T- and B-lymphocyte subset proportions, including increased CD4+ T lymphocytes and decreased B lymphocytes; these exploratory associations require validation in larger cohorts. Radiological findings were predominantly inflammatory, with fibrotic changes infrequently observed, and radiological evolution was generally favorable during follow-up, although treatment regimens were heterogeneous. These preliminary observations contribute to the limited literature on pulmonary involvement in childhood-onset cSS and underscore the need for multicenter prospective studies to confirm these findings and guide early recognition and clinical management.

## Data Availability

The original contributions presented in the study are included in the article/Supplementary Material. Further inquiries can be directed to the corresponding author.
